# Lightweight federated learning for STIs/HIV prediction

**DOI:** 10.1038/s41598-024-56115-0

**Published:** 2024-03-19

**Authors:** Thi Phuoc Van Nguyen, Wencheng Yang, Zhaohui Tang, Xiaoyu Xia, Amy B. Mullens, Judith A. Dean, Yan Li

**Affiliations:** 1 School of Mathematics, Physics and Computing, Centre for Health Research, University of Southern Queensland, Toowoomba Campus, Toowoomba, 4350 QLD Australia; 2 School of Computing Technologies, RMIT University, GPO Box 2476, Melbourne, 3001 VIC, Australia; 3 School of Psychology and Wellbeing, Institute for Resilient Regions, Centre for Health Research, University of Southern Queensland, Ipswich Campus, Ipswich, 4305 Australia; 4 School of Public Health, Faculty of Medicine, The University of Queensland, Herston Road, Brisbane, 4006 QLD Australia

**Keywords:** Biotechnology, Computational biology and bioinformatics, Health care, Biotechnology, Computational biology and bioinformatics, Health care

## Abstract

This paper presents a solution that prioritises high privacy protection and improves communication throughput for predicting the risk of sexually transmissible infections/human immunodeficiency virus (STIs/HIV). The approach utilised Federated Learning (FL) to construct a model from multiple clinics and key stakeholders. FL ensured that only models were shared between clinics, minimising the risk of personal information leakage. Additionally, an algorithm was explored on the FL manager side to construct a global model that aligns with the communication status of the system. Our proposed method introduced Random Forest Federated Learning for assessing the risk of STIs/HIV, incorporating a flexible aggregation process that can be adjusted to accommodate the capacious communication system. Experimental results demonstrated the significant potential of a solution for estimating STIs/HIV risk. In comparison with recent studies, our approach yielded superior results in terms of AUC (0.97) and accuracy ($93\%$). Despite these promising findings, a limitation of the study lies in the experiment for man’s data, due to the self-reported nature of the data and sensitive content. which may be subject to participant bias. Future research could check the performance of the proposed framework in partnership with high-risk populations (e.g., men who have sex with men) to provide a more comprehensive understanding of the proposed framework’s impact and ultimately aim to improve health outcomes/health service optimisation.

## Introduction

Sexually transmitted infections (STIs) and human immunodeficiency virus (HIV) datasets are typically complex and high-dimensional. STIs/HIV data may include socio-demographic information, sexual behaviour, medical history, laboratory test results, etc. In light of the rich and multifaceted nature of STIs/HIV data, encompassing socio-demographic information, sexual behaviour, medical history, laboratory test results, and more, the utilisation of Random Forest (RF) emerges as a highly effective approach. RF was one of the most popular and powerful algorithms to solve unique problems in digital health, including decision-making and predicting actual STIs/HIV infections. RF was a widely used algorithm for predicting STIs/HIV^1–4^, due to its many advantages. RF can effectively handle missing data and categorical variables, which can be common challenges when working with healthcare datasets. Additionally, RF provided valuable estimates of feature importance, aiding in the identification of the most influential factors for predicting the outcome^5^. In addition, RF worked well with high-dimensional data^6^, and a large number of features, which is important for predicting HIV and STIs, as these conditions are often associated with a variety of risk factors, such as demographic, behavioural, and biological^7–9^.

Handling sensitive data like STIs/HIV poses another challenge: the preservation of privacy. In today’s digital age, safeguarding privacy has become increasingly vital in light of rising data breaches and cyberattacks on organisations. This concern is particularly pronounced when dealing with healthcare data and conditions such as STIs/HIV, which carry sensitivity and social stigma. Numerous studies were dedicated to devising methods for safeguarding privacy when analysing sensitive data within a server or centralised repository. These methods encompassed encryption, access controls, and the utilisation of anonymized or pseudonymized data^10^. One approach to protecting privacy was to use data anonymisation and differential privacy techniques to prevent sensitive information from being revealed^11^. Another method involved decentralising data storage and processing to prevent sensitive information from being stored in a single, centralised repository that could be susceptible to being targeted by attackers^12^. FL is a technique for training machine learning models on decentralised data without centralising or sharing the data. Instead, data remains on the device or data centre where they are generated, and only model updates are shared. This approach can protect data privacy and security while allowing for the benefits of shared knowledge and improved model performance^13^. FL distributes the learning process across multiple devices. Therefore, this method reduces the impact of attacks on a single-potential-target data centre. The FL central server only receives models, reducing the risk of data leakage or unauthorised access to the original data.

In healthcare, FL has enormous potential^14^. FL enables the analysis of sensitive medical data without the need for centralisation or data sharing, potentially resulting in discoveries and improved patient outcomes^15^. Integrating electronic personal health records (EHR) from various countries, stakeholders, and federated learning facilitates a greater exchange of information and improves diagnosis and management by clinicians. This combination is essential in diagnosing STIs/HIV as the data related to STIs are susceptible.

Many cultures perceive STIs/HIV as stigmatised conditions^16–18^, including among health professionals^19^ and especially where there is intersectional identity^20–22^. FL allows for the development of models that can generalise across different populations, which is crucial for the prediction of STIs and HIV, which disproportionately affects specific groups, such as men who have sex with men (MSM) and people from higher endemic countries^23^. This can help identify patterns and risk factors that may not be apparent in a single dataset and improve early diagnosis and treatment.

There has been significant research on FL in the field of digital health^13–15,24–26^. However, the application of FL for STIs/HIV prediction remains limited. Certain gaps remain in the application of FL for STIs/HIV risk prediction. To the knowledge of the authors, no one to date has applied FL for STIs/HIV prediction. Even though FL was used in digital health generally, most researchers utilised a fixed configuration to build a global model.

To fulfil this gap, our aims were to explore and create lightweight FL models for STIs/HIV risk prediction. This study also provided a comparison between recent studies on STIs/HIV prediction and our own work. It is evident that all recent studies utilise centralised learning on various types of data, yet they consistently exhibit lower performance than our approach. Our contribution to the current study can be summarised as follows:

- We proposed pre-processing data steps to get prominent features to feed the prediction model.
- We presented a framework that employs RF-based FL to predict the risk of STIs/HIV.
- Additionally, we proposed a novel method for combining models at the server site to decrease the model size and improve the overall system throughput of AI-centric communication systems. The smaller size of an AI model leads to faster response time and fewer computational resources to process and be executed rapidly^27^.
- Moreover, our lightweight AI model needs fewer bits to present its weights/parameters, resulting in a narrower bandwidth requirement for transmitting the model from servers to devices. The AI communication speed can also be increased. A smaller model desires lower energy to execute and transmit. Therefore, power consumption is reduced by scaling down the size of AI models on mobile or edge devices^28^.

The subsequent sections of the paper are structured in the following manner. Section “Federated learning for digital health applications” discusses FL in the digital health domain and compares our work with recent machine learning studies on STIs/HIV prediction. Section “Preliminaries on federated learning versus centralized learning” compares FL and centralised learning. Our proposed framework is presented in section “The proposed framework”. Subsequently, section “Experiment results” presents the experimental results of our framework using a dataset from eight countries. The final section provides a summary and outlines recommended future directions.

## Related works

### Federated learning for digital health applications

In digital health, FL is widespread due to its ability to gather important data from a diverse population without compromising patient privacy. This is achieved by ensuring the data remains on individual devices or clinic centres and never leaves these. Numerous studies have explored the application of the FL in digital health^15,24–26,29–33^.

In 2018, Huang et al.^32^ proposed a method called LoAdaBoost, which utilises hospital intensive care data. This method is based on FL and allows for the communication of model weights and cross-entropy losses between clients and the server. The LoAdaBoost method demonstrated superior results compared to the baseline model—FedAvg model. In the following year, Liu et al.^29^ presented a two-stage federated natural language processing method that allows the utilisation of clinical notes from multiple hospitals or clinics without transferring the data. They demonstrated the performance of this method by using obesity and comorbidities phenotyping as a medical task. This approach improved the quality of a specific clinical task and facilitated knowledge progression in the entire healthcare system, which is an essential part of a learning health system^29^.

The prospects of digital health utilising RF were discussed by N. Rieke and colleagues in detail in reference^15^. Several methods to create a global model were considered in this literature to combine knowledge from multiple data centres. Building a good FL model still faced challenges such as data quality, study protocols/designs, and data acquisition. Additionally, natural biases in digital health data may arise from different brands of medical devices or local demographics, which can lead to lower accuracy of the global FL model on local data. While FL itself does not involve sharing data, it remains important to enhance security measures as the model inversion technique can be used to attain the original data and build machine learning models^34^.

The system architecture presents a challenge when developing an FL system for a digital health application, as each clinic centre must have a reliable and powerful computing system to train the local model. Furthermore, the process of integrating models requires a server or trusted cloud to store local models and combine them into a global model before sending them back to each clinic^15^. The throughput or communication bandwidth of the system is also crucial for transferring and receiving local and global models.

Recently, Rodolfo et al.^14^ published a systematic review on FL for healthcare. They examined FL’s primary research topics and system architecture for digital health. They also emphasised the need for more research on security protection for FL in healthcare to secure sensitive medical data.

Overall, these studies have showcased the application of FL to address various healthcare challenges. However, there remains a gap in providing insights into why FL is superior to centralised learning. We will delve deeper into this issue in the next section of our discussion.

### Preliminaries on federated learning versus centralized learning

AI decision-making is created by building a model from two main approaches: centralised and federated learning. In general, both methods aim to develop good models to solve different problems in the AI area, including prediction and forecasting, recommended systems, fraud detection and so on. Centralised learning (CL) significantly differs from FL in five key respects^35^.

The first difference between CL and FL is about data ownership. In CL, all data are combined to build a prediction model. In digital health, centralised data is essential for patients, doctors, researchers and governments. Many national-level projects of gathering health data from individuals have been approved to help clinicians/researchers enhance prediction outcomes^36^. To give access to and protect this type of data, extensive guidelines/regulations need to be developed. Moreover, centralised data has to deal with several impediments, such as high costs to maintain and protect data, questions around data ownership, and fragmentations. In contrast, FL keeps data on personal devices/computers. The training model process also happens on the devices where data are generated.

Secondly, data privacy is a big issue in the centralised learning system. All data from various clients/users must be sent to the server for analysis and building models. This system’s security and privacy protection must be considered to ensure the whole system is safe from attacks. On the other hand, FL manages their data on their own devices, reducing data leakage problems.

Thirdly, centralised learning requires more frequent communications between clients and servers to ensure the trained model at the server is created from an updated dataset. FL found dramatic differences regarding frequent communication between clients and servers since they only share their model and download the global model from the server. Thus, no raw data needs to be transmitted between clients and central servers. The protocol can be created to maintain communications between clients and servers^37^.

Fourthly, in many cases, centralised learning creates better prediction models than the FL approach since the centralised learning model is trained from all clients’ data. In contrast, models in FL are trained from each client’s limited data^38^. The work by Nilsson et al.^39^ showed FL had similar model performance to centralised learning for independent and identically distributed (i.i.d) data. At the same time, the centralised learning method outperforms FL with non-i.i.d. data.

The last key difference between FL and centralised learning is scalability^40^. FL is suitable for large-scale distributed systems because it can leverage the computational power of millions of devices. In contrast to FL, the centralised learning process may need to be faster or work well with very large data due to server hardware limitations^38^.

After thoroughly reviewing numerous studies on Federated Learning (FL) for digital health, it becomes evident that there is a lack of research focusing on the applications of FL for STIs/HIV risk prediction. Therefore, in this study, we proposed an FL system for STIs/HIV risk prediction. We also investigated a new method to aggregate the global model to enhance throughput and calculation resources for the system.

## The proposed framework

Two main issues must be addressed when developing a federated-learning-based system to predict the output of any given problem. Firstly, the algorithm to build the local model must be carefully selected. Secondly, it is crucial to determine how to aggregate local models to build the global model on the server side. The algorithm used for model aggregation is of utmost importance in optimising the calculation time and throughput of the entire communication system while maintaining a high level of accuracy for the model^41^. Regarding these issues, there seems to be a significant emphasis on addressing STIs/HIV prediction, specifically using tabular data as the model input. Recent literature on the application of machine learning to STIs/HIV prediction shows random forest always produces good results compared with other algorithms^7,9,42–44^. The RF algorithm is considered one of the most popular for questionnaire data since it is a non-parametric algorithm and does not apply or assume distribution for data. In this case, this algorithm is appropriate in case data distribution is unknown or complex. Moreover, RF combines decision trees, and each decision tree works on partitioning a small subset of data. This way of working helps to avoid the problem of outliers in questionnaire data. RF also provides a measure to find essential features, which helps to identify the most relevant feature list to predict the outcomes. For the above reasons, we propose to apply random-forest-based federated learning to solve STIs/HIV risk prediction to enhance the privacy protection of a system as local clinics only share their model to build a global model.

To enhance the efficiency of the global model, we investigated the adaptive aggregation global model based on the available communication resource of the system to enhance the calculation and throughput of communicating links between the server and the client computer. The detail of the proposed system is presented in section  “RFFL for STIs/HIV risk prediction”.

### Pre-processing data

Preprocessing data to feed the AI model is highly important. In this study, the preprocessing steps for predicting STIs/HIV risk incorporate various innovative aspects aimed at obtaining a high-quality dataset and improving the predictive model. A systematic data analysis process, encompassing the handling of missing data, data cleaning, identification and removal of correlated features, and variable importance assessment, is employed to eliminate redundant or less informative features while retaining key factors essential for predicting HIV and STI risks. The utilization of correlation analysis and variable importance scores enhances the precision and efficiency of the predictive modelling approach.

**Figure 1:**
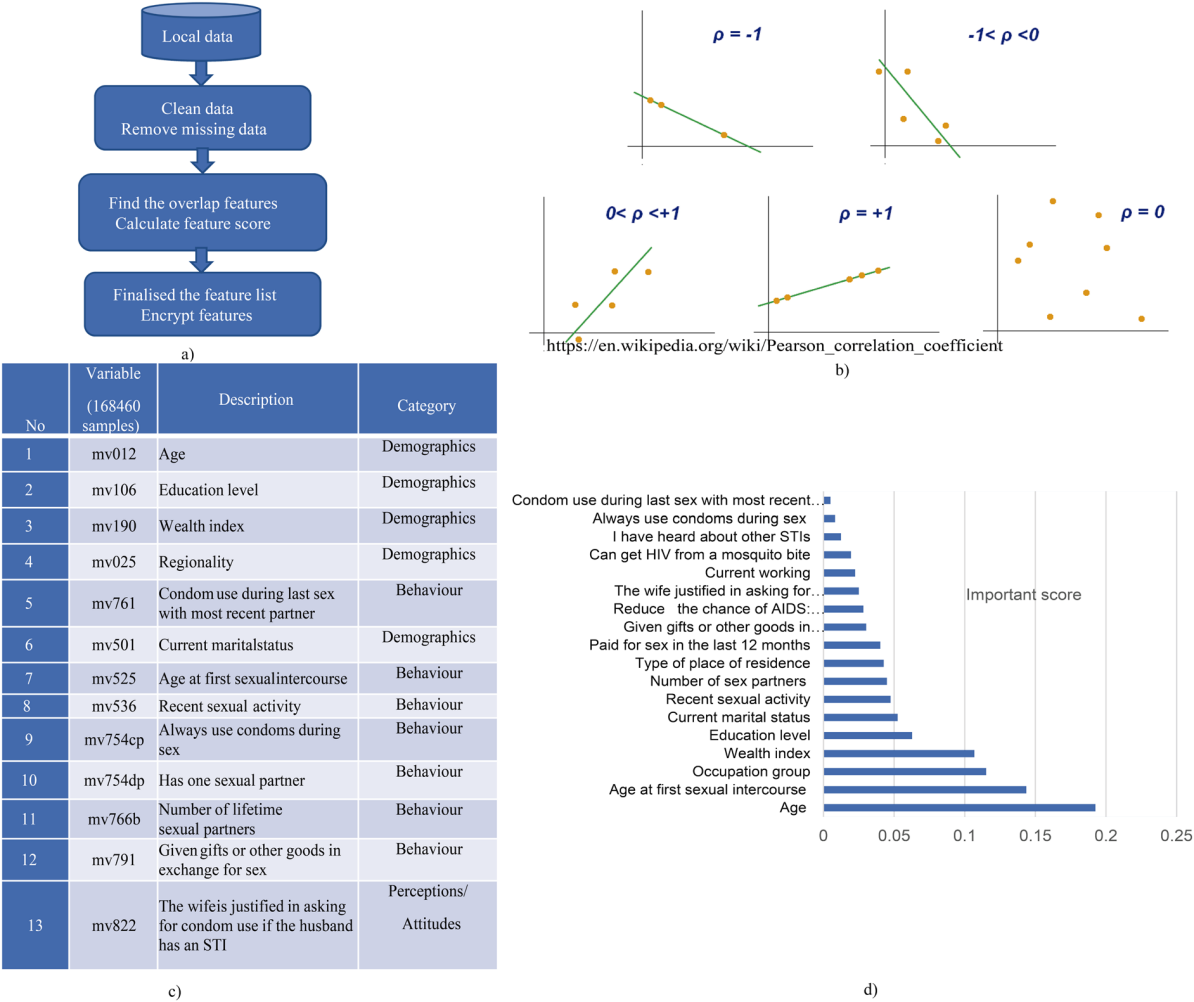
(**a**) Major steps of Pre-processing STI/HIV data; (**b**) Pearson correlation illustration to remove overlap features; (**c**) selected feature/variable list; (**d**) important feature scores.

Our proposed preprocessing data for STI/HIV prediction in each client is illustrated in Fig. 1a.

The initial stage involves preparing a distinct dataset for each client. Once the data have been collected to estimate STIs/HIV risk, cleaning the data, removing missing entries, and addressing any potential issues related to data imbalance are necessary. In this work, the synthetic minority oversampling technique (SMOTE) is used to address the imbalance data issue; the next step is to build a correlation metric to identify overlapping features. The concept of Pearson correlation is explained in Fig.  1b. The Pearson correlation coefficient *r* between two variables $X_i$ and $Y_i$ can be calculated as $r = \frac{\sum {\left( X_i - \bar{X}\right) \left( Y_i - \bar{Y}\right) }}{\sqrt{\sum {\left( X_i - \bar{X}\right) ^2}\sum {\left( Y_i - \bar{Y}\right) ^2}}}$^45^. Ideally, two features will have negative/positive correlations if the Pearson correlation values equal $-1/+1$, respectively. When the absolute value of the Pearson correlation between two features is close to 1, it indicates one of the features can be excluded from the feature list. Subsequently, feature-important scores can be calculated using RF or linear regression algorithms. The feature important scores are illustrated in Fig.  1d. Based on these calculations, the most relevant features are selected to construct the model. Figure  1c listed thirteen important features to build prediction model.

It is important to emphasise the quality of the dataset significantly impacts the accuracy and performance of the model. Furthermore, in this process, we utilise RF, which means that each feature will be presented in the model. The list of features should be encrypted and synchronised for clients.

### RFFL for STIs/HIV risk prediction

**Figure 2:**
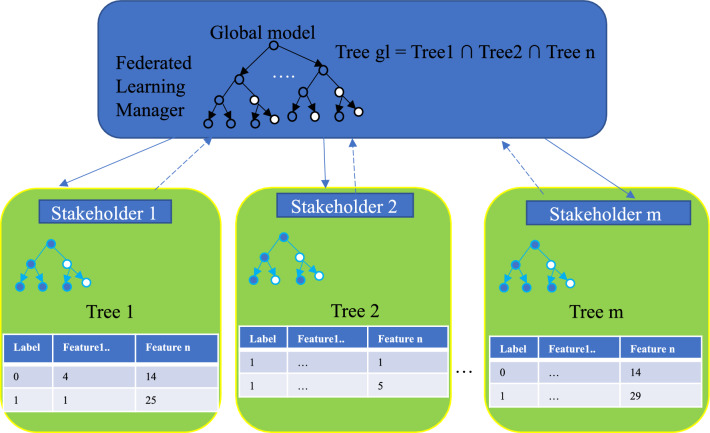
Proposed RFFL for STIs/HIV risk prediction.

Random forest federated learning (RFFL) is a powerful tool for predicting STI/HIV risk, as depicted in Fig.  2. RFFL combines the concepts of RF and FL to address privacy concerns and enhance the accuracy of STIs/HIV risk prediction. By merging the privacy-preserving nature of federated learning with the ensemble learning capabilities of random forests, it enables a collaborative model training while ensuring the protection of sensitive patient data. In this approach, various stakeholders or organisations develop their own models using their respective data, allowing for more diverse and comprehensive modelling of the factors that influence the STIs/HIV risk.

Once each stakeholder has trained their model, these models are then sent to a secure server or trusted cloud that serves as the Federated Learning Manager. The RFFL method then combines the decision trees generated by each stakeholder’s model to create a global tree optimised to balance the communication efficiency between the server and the different stakeholders.

### An enhanced RFFL solution

At the local level, the data is preprocessed to address any imbalances and resolve issues before training, and the resulting local model to solve the binary classification problem (yes or no for HIV/STIs) is saved for sharing purposes. The detailed algorithm for the RFFL system on the server side is presented as follows (Algorithm 1). After receiving the individual models from each client, the server checks the communication throughput between itself and a client. Based on this assessment, the server determines the optimal structure for the global model.

If a communication system has maximum throughput, the server builds the global model using all the client models. However, in a real situation, the FL manager randomly selects a subset of clients, using less than $100\%$ of them, whose models will be utilized to create the global model. This approach ensures that the global model is built with an optimal number of client models, taking into account the limitations of the communication system. In addition, FL managers can build client metrics (data quality, performance of model) and then choose good local models to improve the global model. This model-building process flexibility may help improve the global model and reduce the risk of overfitting. Using an adaptive/flexible approach helps FL process data more efficiently and create a good global model for the system.

Once the global model is built, it is sent back to each client or stakeholder for the STIs/HIV risk prediction. The client can then use this global model to make predictions on their own data and obtain more accurate and comprehensive risk assessments to guide screening.

Overall, this FL approach provides a powerful and secure method for combining the expertise and data of multiple stakeholders to generate more accurate and comprehensive models for STIs/HIV risk prediction. By using this approach, stakeholders can benefit from the advantages of machine learning (high-accuracy model) while ensuring data privacy and security. Further noticed for this system is that each client will encrypt its targeted label values and feature list before training the model. Moreover, the shared model should be built based on common features between all clients.

**Figure:**
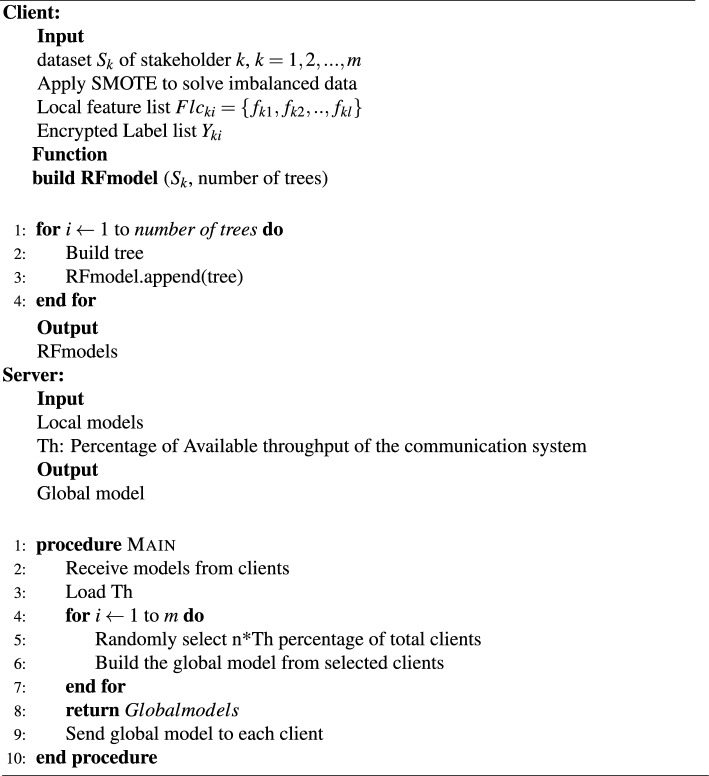
Algorithm 1 Randomly RFFL for STIs/HIV risk prediction

In the next section, the performance of the proposed algorithm is evaluated on the data from eight countries. We consider different percentages of all the client models needed to build the global model. Results for each case of a combination to build the FL model will be compared with local models.

## Experiment results

### Datasets

The study utilised a total of 168,459 records from eight countries. The detail is described in Table 1.

**Table 1:** Datasets summary.

Country	Year	Number of records
Dominican	2013	9717
Dominican Republic	2013	2028
India	2015	107,297
Haiti	2016	9572
Haiti	2012	9202
Guinea	2018	3831
Guinea	2012	3688
Ethiopia	2016	11,327
Cameroon	2018	6648
Angola	2015	50,150

The data from the demographic and health surveys (DHS) Program comprised information on behaviour, clinical testing for HIV/STIs (“yes” or “no” for HIV/STIs), and demographic data for men. The variables were selected through an extensive review of relevant literature^8,9,46^ and consultation with experts in public and sexual health. The research project advisory group includes two of the co-authors (A.Prof. Judith Dean and Prof. Amy Mullens) as we invited key stakeholders (e.g., clinicians, epidemiology/policy and community workers, many of whom are also community members). The project advisory group contributes to co-design consistent with participatory action research to enhance engagement, appropriateness, feasibility and impact.

**Figure 3:**
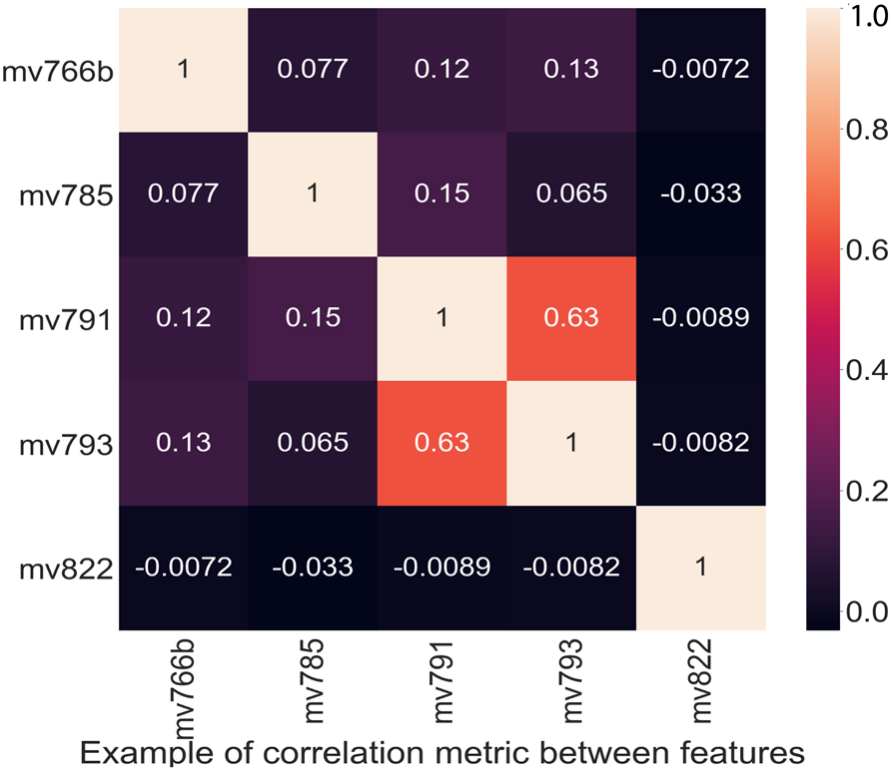
An example of Pearson correlation metric.

**Figure 4:**
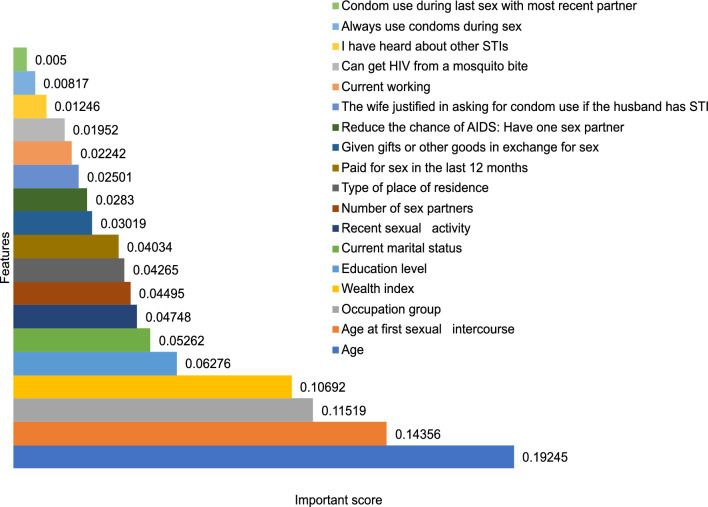
Scored important features for HIV prediction.

The data from each country was collected in a tabular format and underwent preprocessing steps before addressing binary classification for STIs/HIV risk prediction:

- Eliminating missing data: We observed that 94.74% of the data pertaining to the inquiry about seeking advice or treatment for STI infection (No20-mv770) is marked as Nan (Not available or missing data), as numerous countries did not record information for this specific question. As a result, we decided to exclude this variable from our list. In relation to the variables “Ever heard of STIs” (No13-mv750) and “Whether the respondent has ever heard of AIDS1” (No14-mv751), 91% of respondents answered “Yes.” However, these variables offer minimal contribution to a predictive model due to the predominance of the same value across most samples. Consequently, we also opted to eliminate these two variables from our list of variables.
- Removing missing data rows: Each row in this dataset corresponds to information from an individual. Missing data instances may arise when individuals are unwilling to respond to specific survey questions or choose not to answer certain inquiries. Due to the uncertainty surrounding the cause of missing data, a thorough examination was conducted for each country’s dataset, and rows containing NaN values were subsequently excluded. This study adopts the fast and basic method of removing missing observations because the datasets have large sample sizes, ensuring an unbiased and complete dataset, as highlighted by Young’s survey^47^. It’s worth noting that while mean, median, and other imputation methods are suitable for continuous and small datasets, they may not be suitable for categorical or discrete data^48^.
- Calculating Pearson correlation between all features, then removing the overlap features. An example of visualisation for correlation metric is described in Fig.  3, $r=1$ indicates a perfect positive linear relationship, $r = -\,1$ indicates a perfect negative linear relationship, and $r=0$ indicates no linear relationship. We can see that feature coded *mv*791 (Ever provided gifts or other goods in exchange for sex) and feature *mv*793 (Paid for sex in the last 12 months) have a high correlation so that we can exclude one from the feature list.
- Scoring feature importance by the RF algorithm and then choosing the most important feature list. The feature important score for the HIV prediction is illustrated in Fig. 4. This score evaluation allows us to choose the feature list to build the final global model.
- Encrypt feature list

Our final local model was trained with 13 features, including *age, education level, wealth index, regionality, condom use during last sex with a most recent partner, current marital status, age at first sexual intercourse, recent sexual activity, always use condoms during sex, have one sexual partner, number of lifetime sexual partner, given gifts or other goods in exchange for sex, and the wife is justified in asking for condom use if the husband has an STI.*

### Model selection

The optimal hyperparameters in the RF model can vary based on the dataset and the specific problem to be addressed^49^. Increasing the number of trees ($n_estimators$) tends to enhance the model’s accuracy. However, there is a threshold beyond which additional trees may yield little improvements^50^. After conducting experiments with our datasets, we carefully selected relatively conservative hyperparameters for the RF model, including a small number of estimators ($n_estimators$ $=5$) and a minimum number of samples per leaf ($min_samples_leaf$ $=3$), for each client/stakeholder involved in the study.

### Evaluation metrics

To check the performance of the proposed method, we use two metrics, area under the receiver operating characteristic curve (AUC) and accuracy. The AUC is determined by approximating the area below the receiver operating characteristic (ROC) curve. The ROC curve displays the relationship between the true positive rate (TPR) and false positive rate (FPR) for various thresholds applied to the classifier. In medical applications, a model with AUC larger than 0.9 is considered an excellent model^51^.

The second metric used for this work is accuracy which is calculated as follows:

1 $$\begin{aligned} Accuracy = \frac{TPN+TNN}{TN} \end{aligned}$$

where TPN is the true positive number, TNN is the true negative number, and TN is the total number.

### Results

To showcase the efficiency of our proposed system, we perform various levels of aggregation to construct a global model on the server side. FL with $100\%$ of aggregated means we aggregated all client’s models to build the global model. FL with $10\%$ of aggregated means only $10\%$ of client models are used to build the global model. The capacity of the global model is then calculated for each case. Next, we analyse the relationship between the model performance and the capacity to develop guidelines for the system. The performance of the proposed system and comparison with the recent work (Xu et al.^7^) and the FL CNN model are summarised in Table  2.

Notably, the FL model outperforms the local models, as evidenced by its higher AUC and accuracy scores. Additionally, even when the capacity of the global model is reduced by $30\%$, the AUC and accuracy values of the FL model remain high, similar to those of a $100\%$ aggregated model. This indicates that the system’s throughput can be improved by reducing the data transfer from server to client. Moreover, our proposed system showed outperforming results from federated learning convolutional neural network (FLCNN) and Xu et al.^7^.

**Table 2:** Performance of Random Forest Federated Learning with different levels of model aggregation.

Models	HIV prediction			STI prediction		
	Global model capacity	AUC	Accuracy	Global model capacity	AUC	Accuracy
Local model		0.89	0.82		0.96	0.92
FL with 100% aggregated	4.36 MB	0.91	0.84	2.28 MB	0.98	0.94
FL with 70% aggregated	3.25 MB	0.91	0.83	1.67 MB	0.98	0.93
FL with 50% aggregated	1.75 MB	0.90	0.84	1.09 MB	0.97	0.93
FL with 30% aggregated	1.42 MB	0.90	0.83	840 KB	0.97	0.93
FL with 20% aggregated	769 KB	0.90	0.83	508 KB	0.97	0.93
FL with 10% aggregated	348 KB	0.89	0.82	345 KB	0.96	0.92
FLCNN	964 MB	0.87	0.78	964 MB	0.74	0.70
Xu et al.^7^	NA	0.72	NA	NA	0.67–0.75	NA

**Figure 5:**
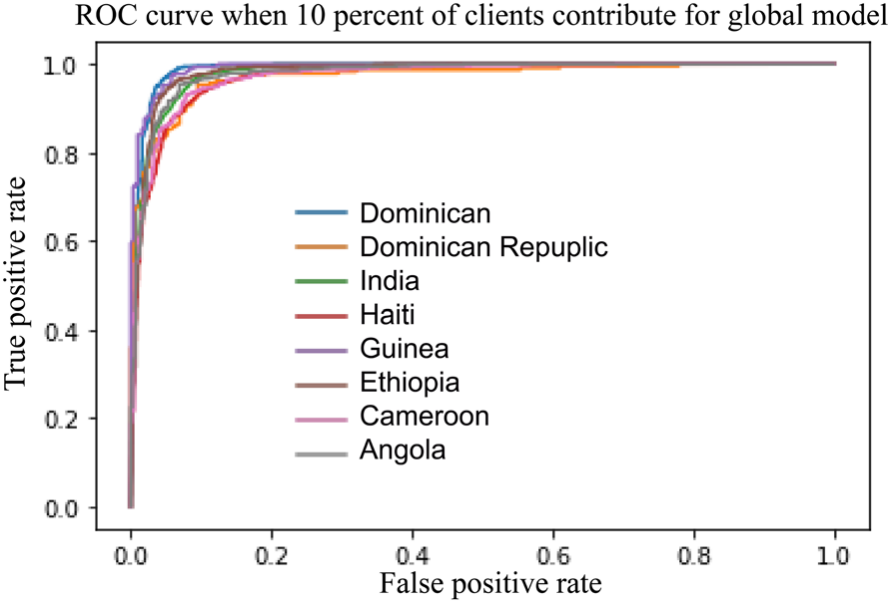
Model performance of 100% aggregation to predict HIV.

**Figure 6:**
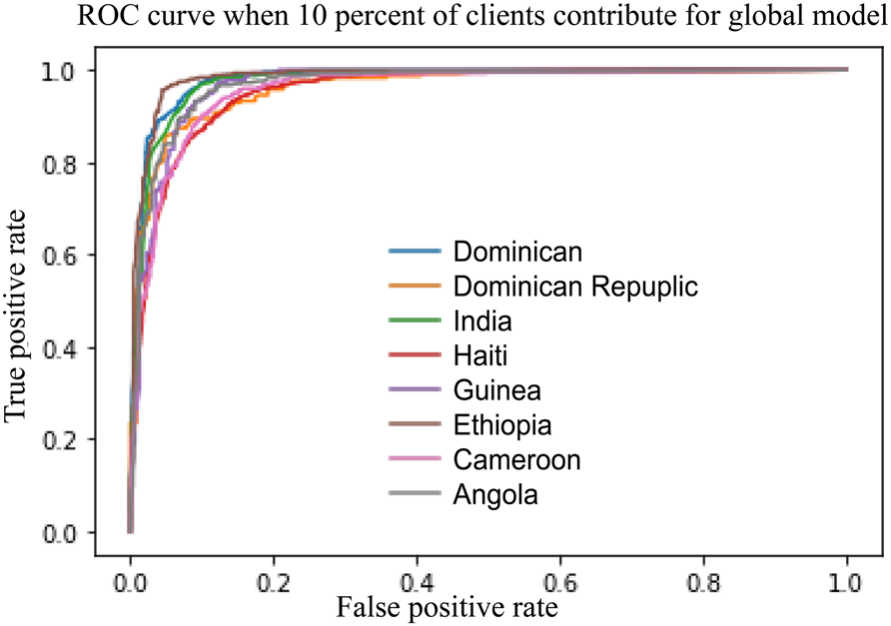
Model performance of 10% aggregation to predict HIV.

The ROC curve illustrates the performance of a binary classifier system, as shown in Figs.  5 and 6, which represent data from 8 countries when applying $100\%$ and $10\%$ model aggregation, respectively. The global model’s performance for the Dominican and Dominican Republic is outstanding, achieving AUC values of 0.97 and 0.96 (for $100\%$ aggregation) and 0.94 (for $10\%$ aggregation). Conversely, India and Haiti exhibit lower performance, with AUC values of 0.8 (for $100\%$ aggregation) and 0.78 (for $10\%$ aggregation). The remaining countries display AUC values ranging from 0.96 to 0.8 for both aggregation scenarios. Notably, despite the reduced level of aggregation, the quality of the model remains robust, indicating the effectiveness of our proposed solution even under constrained communication throughput.

## Discussion

In previous studies, some have focused on AI predictions for both STIs and HIV, while others concentrated solely on HIV or STIs. To ensure a fair comparison, we assessed the maximum accuracy and AUC for HIV or STI risk prediction in our work against the findings of previous studies. When compared to the other studies listed in Table  3, our work adds a novel contribution by achieving the highest maximum AUC of 0.97 and an accuracy of $93\%$. These performance metrics clearly indicate that our proposed RFFL approach for assessing STIs/HIV risk outperformed the other studies regarding predictive accuracy.

Furthermore, our work introduces the RFFL approach, which offers a unique contribution by enabling an adaptable aggregation process aligned with the throughput of the communication system. This adaptability is crucial in FL, where data remain decentralised, and efficient aggregation methods are necessary to ensure accurate and timely model updates. In resource-constrained healthcare settings, efficient model updates are crucial. RFFL’s ability to align with the throughput of the communication system minimises resource usage. This is especially beneficial in low-resource areas with limited bandwidth and computational resources.

**Table 3:** The comparison of the proposed method and the existing studies on machine learning application deployment for STIs or HIV prediction.

Paper	Year	Max AUC	Max accuracy	Highlights contribution	Type of learning
Ahlström et al.^52^	2019	0.89	N/A	This study demonstrates the feasibility of utilising machine learning techniques, and centralised data to accurately predict an individual’s HIV status based on an electronic registry data	Centralized learning
Xu et al.^7^	2022	0.75	N/A	Authors developed a machine learning-based risk-prediction tool for STI/HIV. This tool can be integrated with digital platforms to increase STI/HIV testing	Centralized learning
Bao et al.^8^	2021	0.76	82%	This research demonstrated the advantage of machine learning over the logistic regression model on Australian Man Who has sex with a man group	Centralized learning
Balzer et al.^46^	2020	0.73	N/A	They used super learner—a type of machine learning model to demonstrate the potential of using AI in evaluating HIV risk score	Centralized learning
Our work	2023	0.97	93%	We proposed a RFFL approach to assess the STIs/HIV risk. The framework allows for an adaptable aggregation process that can align with the throughput of the communication system	Federated learning

The comparative analysis reveals that while other studies have explored using machine learning techniques for STIs/HIV prediction, they achieved lower maximum AUC values ranging from 0.73 to 0.89. Additionally, some studies focused on centralised learning^8,46^, whereas our work utilised FL, which has advantages in preserving data privacy and enabling collaboration among multiple clients.

The significantly higher maximum AUC and accuracy obtained in our work demonstrates the superiority of the proposed RFFL approach. These results strongly indicate our approach is most effective in capturing the complex relationships and patterns in the data, leading to improved predictions of STIs/HIV risk.

It is important to note that selecting the “best” solution depends on various factors, including the specific context, dataset characteristics, and evaluation criteria. However, based on the provided performance metrics and the unique contribution of our RFFL approach, our work demonstrates exceptional performance compared to the other studies listed in Table  3.

## Conclusion

In this study, we explored the application of RFFL with demographic and behaviour data to predict the risk of HIV and STIs. RFFL’s adaptable aggregation minimises the amount of data that needs to be shared across different healthcare entities. This aligns with the privacy-preserving characteristics of FL, reducing the risk of data breaches and ensuring that patient information is protected throughout the model training process. The proposed method leverages global aggregation based on the communication system throughput to achieve optimised results. Our results demonstrated the significant potential of RFFL and global aggregation for practical use in this domain.

We used demographic and behaviour data to train and test the RFFL model and compared its performance with existing models. Our experiments demonstrate that the RFFL outperforms the other models’ accuracy and AUC by $10\%$. Additionally, we tested the proposed method of global aggregation based on communication system throughput and found that it effectively balances the model performance and communication efficiency.

The results of our study demonstrated the potential of the RFFL and global aggregation for predicting the risk of HIV and STIs using demographic and behavioural data. The proposed method had implications for practical applications in the healthcare industry, such as the advantages of early treatment of patients. It can be used as a model for similar predictive tasks.

This work can be extended to optimise the selection of client model processes to build a global model. The selection of clients can be conducted in a way that ensures that the diversity of data in the global model is maintained. For example, the FL manager selects clients with different data types or data from different geographical locations. Furthermore, the local model quality metrics should be taken into account to build a global model.

With these advancements, our research laid a strong foundation for revolutionising STI/HIV prediction. It presented a promising path towards improving healthcare outcomes and fostering data-driven innovation in the future.

## Data Availability

The dataset used in this paper was obtained from The Demographic and Health Surveys (DHS) Program (https://dhsprogram.com/Data/terms-of-use.cfm) with permission granted for the project titled “AI Assistant to Predict HIV STI,” approved on April 19, 2022.
